# The UK Maternity Crisis: Analysing the Underlying Causes to Find Solutions

**DOI:** 10.1111/1471-0528.18326

**Published:** 2025-08-12

**Authors:** Andrew D. Weeks, Sarah Espenhahn, Susie Crowe

**Affiliations:** ^1^ University of Liverpool/Liverpool Women's Hospital Liverpool UK; ^2^ NHS England London Region London UK; ^3^ British Intrapartum Society London UK; ^4^ Barts Health NHS Trust London UK

**Keywords:** delivery: birth trauma, delivery: caesarean section, health services research, labour: management, maternity services, medical law

Lord Darzi, in his recent report, concludes that ‘too many women, babies and families are being let down’ by UK maternity services [1]. Complex underlying factors have put UK maternity units under significant pressure with repeated reports of poor work cultures, over‐stressed staff leaving the NHS, stories of birth trauma and calls for a national maternity inquiry. Whilst maternal and perinatal outcomes are significantly better than those in the United States [2], they lag behind those in many comparator countries in Scandinavia and the rest of Europe [3]. Whilst the proportion of obstetric related negligence claims sits at around 10% of the total, the costs of maternity negligence payments are soaring and at £1.1 billion per year are over a third of the total UK maternity budget [4]. The perception might be that standards have fallen and that outcomes are worsening. But despite decreasing births rates, whole time equivalent doctors and midwives have been increasing for many years [5], and term stillbirth and neonatal mortality and morbidity rates are steadily improving [6]. Judging by the most commonly used important outcome, perinatal mortality, you could argue that the standard of care has never been better. So, why does UK maternity care appear to be in crisis?

First, there is increasing medicalisation of birth caused by multiple interrelated factors (Figure 1). Pregnant women in the UK are becoming older, increasingly overweight, have more complex medical problems—all risk factors for adverse outcomes. The increased ability of fetal medicine to detect fetal abnormalities and identify women as ‘high risk’ mean that more parents are approaching birth with anxieties about the outcome. Meanwhile, recent studies have found that induction of labour can reduce many adverse medical outcomes, not least by preventing stillbirths [7, 8, 9]. Combining this with the national ambition around maternity safety [10] and the legal requirement to inform women of all options that can reduce stillbirth [11], means that many practitioners and women feel pressurised into labour induction. The increase in induction rates (to 33% nationally [12]) has led to delays [13] and poor experience, resulting in more women opting for a caesarean birth instead. The NHS maternity staffing and estate, designed to support high numbers of ‘low risk’ births, has yet to fully adapt to the increased numbers on ‘high risk’ care pathways, further exacerbating the problem.

**FIGURE 1:**
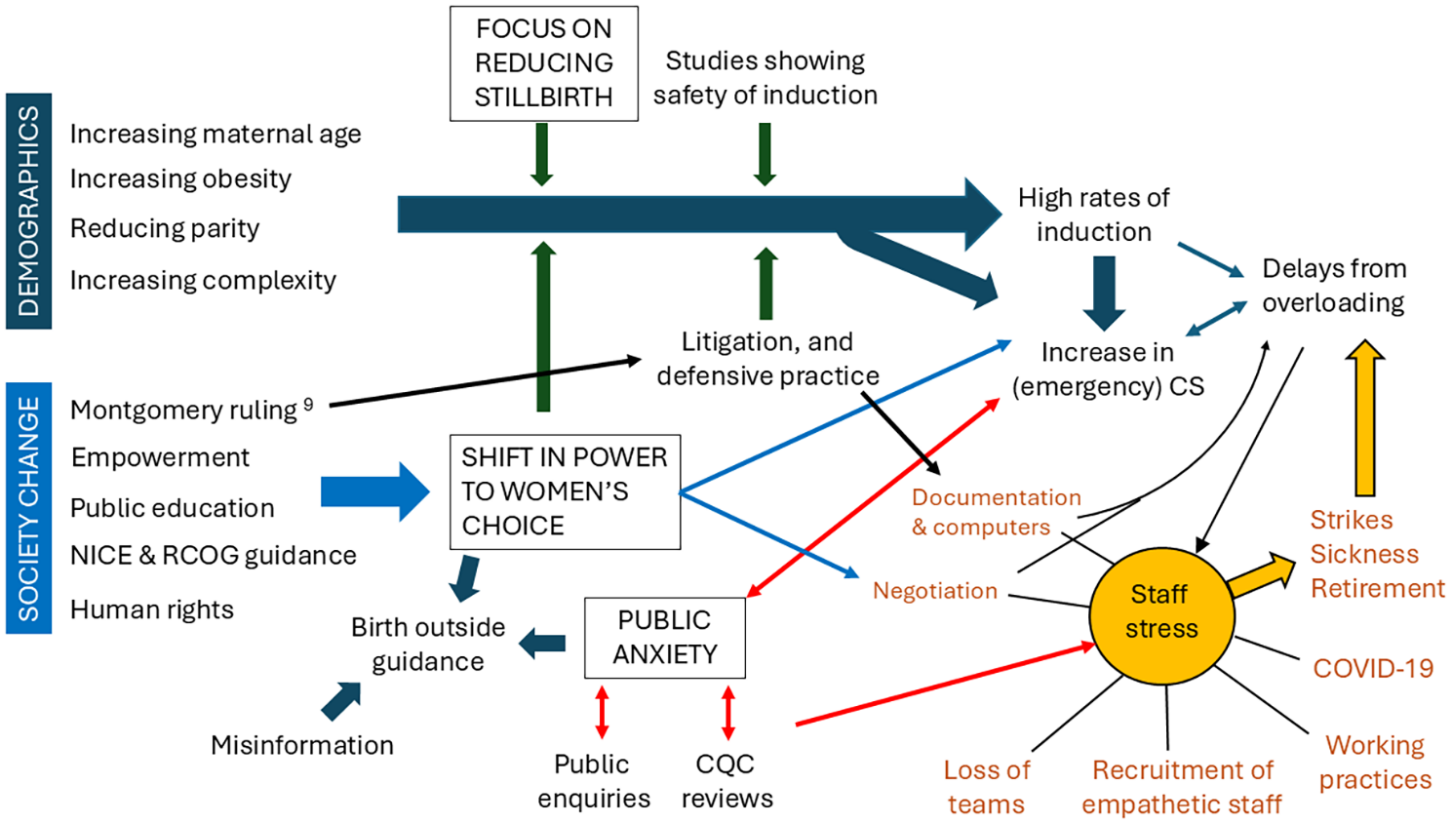
Some of the interlinked factors currently affecting the UK maternity system. [NICE, National Institute for Health and Care Excellence; RCOG, Royal College of Obstetricians and Gynaecologists; CS, Caesarean section; CQC, Care Quality Commission; COVID‐19, Coronavirus disease 2019].

Second, there is a shift in who controls birth. Traditionally, providers have adopted a very medical model in which the doctor was in charge, and this remains the case in societies with marked social hierarchies or in specialisms that deal with acute specialist pathologies such as oncology or general surgery. But society has moved on. The information revolution means that doctors are not the only ones who possess the power of knowledge. Guidelines are widely available online and a quick internet search will provide you with much of the information you need. Women and families get their information from a variety of sources, with a growing reliance on social media platforms. So maternity staff are increasingly there to help interpret information and facilitate care pathways. The shift towards patient power is supported by institutions such as NICE and the Royal Colleges, backed up by the Montgomery legal case [11]. The debate is no longer about whether doctors or midwives should decide women's care: it is rightly now women who should be in control of their own care. However, the system is not yet mature enough to support this, and not every maternity unit and practitioner has adopted it. The Montgomery requirement to offer caesarean birth antenatally, as well as in urgent situations such as late second stage, can easily be taken to imply that caesarean is not only safe but advisable in these situations—further driving up intervention rates. But this is a major change in maternity culture and has not happened everywhere, and many women continue to be subjected to care that they do not wish to have (either over‐ or under‐intervention) leaving them feeling unheard. Parents' anger from this is reflected in calls for national inquiries.

The inquiries into maternity care in Shrewsbury, Morecambe Bay, East Kent and Nottingham have created a national focus on birth safety. Perhaps with the knowledge that those units under the spotlight are not those with the poorest outcomes nationally [14], the inquiries' recommendations have been wide‐ranging and sought to accelerate the national move to woman‐centred, high‐quality care. However, whilst well‐intentioned, their public message that maternity services are failing and dangerous has also caused harm by creating significant public distrust and by reducing staff morale leading to problems of recruitment and retention.

Providing woman‐led care sounds straightforward but is not easy to enact in a public health system. Historically, there was general agreement between professionals as to what level of maternity intervention was appropriate, with the safety of the mother largely placed above that of the baby. This unspoken principle originated in times of high fertility rates, where mothers' ability to reproduce again was prioritised, even if it came with increases in fetal risk. Yet, when the choices are put to mothers, most now prioritise the baby's health over their own safety and choose an interventionalist approach. Others have different priorities and choose management that falls outside of current evidence‐based practice. Guidelines, based on population norms, attempt to provide a detached, logical, risk–benefit analysis. But in deciding for yourself, this logic generally comes secondary to individual considerations such as past negative experiences of care, personal fears (exacerbated by official reports of a maternity crisis) and a desire for control in paternalistic maternity systems. Unsurprisingly, the overall effect is a diversification of birth pathways, with increases in both medicalisation (caesarean birth and induction of labour) and physiological births, even in untraditional groups like twins and other complex pregnancies.

Finally, all the above issues require increased numbers of staff, ideally providing continuity of care. Even in a conveyor‐belt, ‘one‐size fits all’ maternity system, it takes large numbers of expert staff to provide high‐quality care. But personalised care with informed maternal decision‐making increases the time needed for consultations. The staff doing the counselling not only need to know what best practice is, but also the evidence and risks for a wide range of alternative options—and have the time and skills to work through them with women. And, given the high risk of litigation, detailed notes about exactly what was told to the woman need to be typed into new (but sometimes clunky) computerised patient records. This all takes a lot of training and time, and reduces the capacity to speak to those who are more vulnerable. A prolonged antenatal appointment counselling an articulate and empowered woman carrying twins who wants a home birth can easily leave the non‐English speaking 40‐year‐old with diabetes and hypertension lacking time for the necessary personalised care.

Balancing the need to adhere to clinical guidelines in order to optimise safety whilst providing the care that women choose can cause considerable stress when they are incompatible. If you add that to the implicit stress of the role of the obstetrician, and intensive internal and external retrospective scrutiny of complex dynamic decision‐making, then the high levels of burnout and sickness come as no great surprise [15]. Many staff report an increasing loss of psychological safety.

The above analysis may seem complex and leave individual clinicians feeling hopeless about how to address it. Indeed, there are many factors that will either not change, will exacerbate with time (e.g., the demographic changes) or that should be welcomed (e.g., the shift in power to women's choice). Despite these changes, even within a highly pressurised system, it remains possible to deliver compassionate, personalised care that ensures women feel safe, listened to and supported to make the choice that is right for them and their baby. However, it is clear there are systemic barriers to providing this consistently, and these need to be addressed both locally and by those developing national strategy and policy.

First, when the above analysis has been presented nationally, many clinicians seem relieved to see a framework that explains why they feel under such great pressure. Many have described moral injury in the current situation; they cannot provide the standard of care that they would like due to the time constraints on each contact within the current system, which in turn drives inequity of outcomes. Second, there are some system stresses that are caused by generational change and should pass as a new cohort of clinicians are trained. Staff trained in the last century or in cultures outside of the UK may have very different social constructs for care that do not recognise the centrality of women's choice or do not know how to safely implement it. This can be a source of considerable friction in consultations and can result either directly or indirectly in women birthing without medical input or opting for no care at all rather than being subjected to what they consider to be controlling or coercive care. In addition to more staff training, there should be a comprehensive national review of antenatal care models which take into consideration the complexity of consultations and need for specialist input. This would support more personalised, equitable care.

Third, it is clear that in some cases, maternity governance processes have not been providing compassionate care to families who report compounded harm. They are also not creating cultures in which staff feel safe to raise concerns, and this restricts opportunities to learn and prevent unsafe practices. A review of the current landscape with a focus on trauma‐informed care would help to create a culture of learning that supports families and staff alike.

The difficulties with litigation and defensive practice are less easy to address, but a system of no‐fault compensation as used elsewhere in the world may provide a solution. The need for detailed computerised documentation will remain, but advances in technology will make this less labourious as automated voice transcription reduces the need for typing, and the sharing of consultation transcripts with women improves communication and thus informed consent.

Sadly, it could take many years before the above have a widespread effect. Furthermore, they will not occur without a significant increase in funding as well as a shift in focus. Woman‐led, personalised care and high levels of intervention are expensive, and significant changes in staffing models are required to support it.

## Author Contributions

A.D.W. had the original idea which was then developed in discussion with S.E. and S.C. A.D.W. then wrote the first draft of the manuscript which was edited by S.E. and S.C. All authors approved the final version before publication.

## Conflicts of Interest

The authors declare no conflicts of interest.

## Data Availability

The authors have nothing to report.
